# Anhedonia and its sub‐component processes predict clinically significant symptoms of Major Depressive Disorder (MDD) and loneliness in young people

**DOI:** 10.1111/bjc.70008

**Published:** 2025-08-14

**Authors:** Katie Prizeman, Ciara McCabe

**Affiliations:** ^1^ School of Psychology and Clinical Language Sciences University of Reading Reading UK

**Keywords:** anhedonia, loneliness, longitudinal, Major Depressive Disorder, mental health, psychometrics, reward processing, transdiagnostic, young people

## Abstract

**Objectives:**

Anhedonia, a core symptom of Major Depressive Disorder (MDD), is a risk factor for future depressive episodes and is associated with social withdrawal, which may contribute to loneliness—another risk factor for depression. Understanding how anhedonia and its sub‐component processes relate to depression and loneliness could reveal key targets for intervention development.

**Methods:**

We recruited 275 young people (*M*_age_: 20.50) with clinically significant symptoms of depression, indicated by scores ≥27 on the Mood and Feelings Questionnaire (MFQ). Participants completed the Anhedonia Scale for Adolescents (ASA) and its three subscales: ASA‐S1 (Enjoyment, Excitement, and Emotional Flattening); ASA‐S2 (Enthusiasm, Connection, and Purpose); ASA‐S3 (Effort, Motivation, and Drive); and the UCLA Loneliness Scale (UCLA) at baseline and at four‐month follow‐up (N = 173). Multiple regression analyses examined the relationships between anhedonia, MDD, and loneliness, both cross‐sectionally and longitudinally.

**Results:**

Cross‐sectionally, the ASA total scores (β = .655, *p* < .001), ASA‐S1 (β = .586, *p* < .001), and ASA‐S3 (β = .153, *p* = .034) were associated with MDD. ASA total scores (β = .651, *p* < .001), ASA‐S1 (β = .397, *p* < .001), ASA‐S2 (β = .196, *p* < .001), and ASA‐S3 (β = .176, *p* = .018) were associated with loneliness. Longitudinally, ASA total scores (β = .485, *p* < .001) and ASA‐S1 (β = .298, *p* = .008) predicted MDD, while ASA‐S2 showed a trend toward predicting loneliness (β = .099, *p* = .058).

**Conclusions:**

This study highlights how specific anhedonia sub‐component processes predict increases in clinically significant symptoms of MDD and loneliness among young people, informing the development of more targeted treatments for anhedonia.

**Practitioner points**

- Research has linked anhedonia to social withdrawal, loneliness, and Major Depressive Disorder (MDD), all of which are associated with an increased risk of recurrent depressive episodes.
- This study examined how anhedonia and its sub‐components, measured using the Anhedonia Scale for Adolescents (ASA), relate to clinically significant symptoms of MDD and loneliness in young people over time.
- Our findings indicate that anhedonia and its sub‐component processes predict increases in MDD over time and potentially in loneliness as well.
- Identifying the specific anhedonia sub‐components that predict increases in MDD and loneliness may inform the development of more targeted anhedonia treatments.

## INTRODUCTION

Major Depressive Disorder (MDD) is a leading cause of illness and disability in adults and young people (Polanczyk et al., 2015). MDD is a multifaceted disorder that encompasses a variety of symptoms, including persistent sadness, fatigue, changes in appetite or sleep, feelings of guilt or worthlessness, difficulties concentrating, and anhedonia—the loss of interest and pleasure (APA, 2022). The age range of 17–25 years represents a critical developmental period characterised by rapid biological, psychological, and social changes. During this time, brain maturation—especially in the prefrontal cortex—plays a vital role in emotional regulation, decision‐making, and impulse control (Casey et al., 2008). Psychologically, individuals consolidate identity, autonomy, and self‐concept, often alongside heightened emotional sensitivity and vulnerability to stress (Prizeman et al., 2023; Steinberg, 2005). Socially, young people navigate evolving relationships, family dynamics, and increasing independence (Arnett, 2000, 2007).

MDD during this stage can disrupt these developmental processes, interfering with identity formation and increasing the risk of social withdrawal and loneliness (Kemph, 1969; Steinberg, 2005; 2024). Anhedonia may further exacerbate these effects by reducing motivation to seek rewarding or meaningful experiences, thereby limiting opportunities for personal growth, social connection, and recovery (Treadway & Zald, 2011). Early‐onset MDD is associated with long‐term negative effects on health, economic, and social outcomes (Clayborne et al., 2019; Hawton et al., 2012; Prizeman et al., 2025) and predicts high rates of recurrence during adulthood (Dooley & Fitzgerald, 2012; Dunn & Goodyer, 2006). As current treatments are only moderately effective, the development of novel interventions for MDD in young people is a priority (Thapar et al., 2022). It is thought that focusing on certain symptoms, such as anhedonia, which can predict MDD, may enhance the development of treatments (Forbes & Dahl, 2012; Ma et al., 2025; McCabe, 2018).

Anhedonia is a transdiagnostic symptom featuring in MDD, schizophrenia, substance misuse, and Post‐Traumatic Stress Disorder (PTSD) (APA, 2013). Estimates indicate that between 50% and 70% of individuals with MDD experience anhedonia (Goodyer et al., 2017; Orchard et al., 2017; Serretti, 2023). It is widely considered a risk factor for MDD (Hasler et al., 2004; McCabe, 2014; McMakin et al., 2012) and predicts worse depression treatment outcomes (McMakin et al., 2012; Uher et al., 2012).

The Cognitive‐Behavioural Model of Depression suggests that a lack of positive reinforcement from pleasurable experiences can exacerbate depressive symptoms and social disengagement (Brown, 2001; Lewinsohn et al., 1998), and the Social Signal Processing Theory suggests a diminished ability to experience pleasure may lead individuals to withdraw from social interactions, intensifying feelings of loneliness (Cacioppo et al., 2006; Hawkley & Cacioppo, 2010). More recently, researchers have been applying theoretical models of anhedonia based on the neurobiology of reward, suggesting that anhedonia could be underpinned by dysfunction in any one or combination of reward sub‐component processes: anticipation, motivation, and enjoyment (Ma et al., 2025). In line with this, longitudinal studies show that anticipatory anhedonia at one month mediated the relationship between fear of positive evaluation at baseline and depressive symptoms three months later (Jordan et al., 2018), leading the authors to conclude that fearing positive evaluation leads to an inability to look forward to positive events, which in turn leads to the development of other depressive symptoms. Physical anhedonia has also been associated with depressive symptoms in depressed patients assessed six times over 20 years, highlighting that anhedonia may indicate a more severe or persistent form of the illness (Shankman et al., 2010). Given these findings, we argue that a more detailed assessment of anhedonia, including its sub‐component processes as predictors of MDD over time, is needed, as this could aid the development of novel effective reward‐based interventions (Ma et al., 2025).

Therefore, the aim of this study was to assess the relationship between anhedonia and MDD using the recently established Anhedonia Scale for Adolescents (ASA) (Watson et al., 2021). This scale was developed to examine the anhedonic sub‐component processes that correspond to the neurobiological aspects of reward function. It was created through a rigorous, quantitative process: first piloted in 66 adolescents, then an exploratory factor analysis (EFA) was conducted in 1,057 participants, followed by a confirmatory factor analysis (CFA) conducted in an independent sample of 1,041 adolescents (Watson et al., 2021). The final measure comprises three subscales: ASA‐S1 (Enjoyment, Excitement, and Emotional Flattening); ASA‐S2 (Enthusiasm, Connection, and Purpose); and ASA‐S3 (Effort, Motivation, and Drive). These subscales closely align with the reward sub‐components of consummatory, anticipatory, and motivational processing. The ASA significantly predicted elevated depression symptoms [high vs. low Mood and Feelings Questionnaire (MFQ) scores], above and beyond other measures of anhedonia and pleasure (Watson et al., 2021).

Previous studies have also demonstrated that anhedonia is associated with greater levels of loneliness in young people (Barkus & Badcock, 2019; Goldstein et al., 2021), and we have shown that social anhedonia is linked to reduced motivation for prosocial behaviour in MDD (Setterfield et al., 2016). We suggest this may partly underpin impairments in social functioning (Kupferberg et al., 2016; Saris et al., 2017), leading to social withdrawal and ultimately loneliness in MDD. Consistent with this notion, we recently found that young people with high depression symptoms experienced a reduced quality and quantity of social interactions, along with diminished motivation to engage in real‐life social activities (Frey & McCabe, 2020). We have also recently shown deficits in anticipatory pleasure predicting social activities in young people with depression compared to controls using real‐life ecological momentary assessments (Sahni & McCabe, 2025). However, to date, no studies have directly examined how anhedonia and its sub‐component processes relate to loneliness in young people, above and beyond depressive symptoms, or whether anhedonia predicts loneliness over time.

Given these gaps in the literature, the aim of this study was to examine anhedonia and its sub‐components, as measured by the ASA, and their association with depression and loneliness at baseline, as well as at a four‐month follow‐up, in adolescents and emerging adults exhibiting clinically significant symptoms of MDD. In this study, we use the term ‘young people’ to refer to individuals aged 17–25 who met the clinical threshold for depressive symptoms, as defined by a score of 27 or above on the MFQ.

We particularly adopted a symptom‐based approach, assessing depressive symptoms dimensionally and including all participants based on their MFQ scores. This allowed us to capture young people who may be undiagnosed or not currently engaged with services, yet who experience clinically significant levels of distress and impairment. This approach is increasingly recognised as crucial in identifying at‐risk individuals early, particularly in youth populations where symptoms may be emerging, fluctuating, or subthreshold but still cause significant difficulties (Jensen‐Doss & Weisz, 2008). As such, our sample reflects a population highly relevant to mental health services and early intervention efforts, aligning with calls for more inclusive and transdiagnostic research frameworks.

Specifically, we sought to investigate whether higher levels of anhedonia at baseline would predict greater depression and loneliness both at the initial timepoint (Time 1; T1) and four months later (Time 2; T2). Our hypotheses were: (i) at baseline (T1), higher anhedonia total scores and subscale scores would be associated with higher levels of MDD and loneliness, controlling for ethnicity and gender; and (ii) at baseline (T1), higher anhedonia total scores and subscale scores would predict increased MDD and loneliness symptoms at four‐month follow‐up (T2), controlling for ethnicity, gender, and T1 symptom measures.

## METHODS

### Data, materials, code, and online resources

Data were collected exclusively for this study and are publicly available in de‐identified form via the University of Reading's Research Data Archive. Full details of measures, exclusions, sample size decisions, and procedures are reported.

### Ethical considerations

All procedures contributing to this work comply with the ethical standards of the relevant national and institutional committees on human experimentation and with the Declaration of Helsinki (1975), as revised in 2008. The study was approved by the Research Ethics Committee (REC) (reference number: 2022‐072‐NW) of the University of Reading's Psychology Department, and written informed consent was obtained from all participants. Participants received a debrief form, which included contact details for the Samaritans and advised anyone concerned about their mood to contact their general practitioner (GP).

### Participant eligibility criteria

**FIGURE 1:**
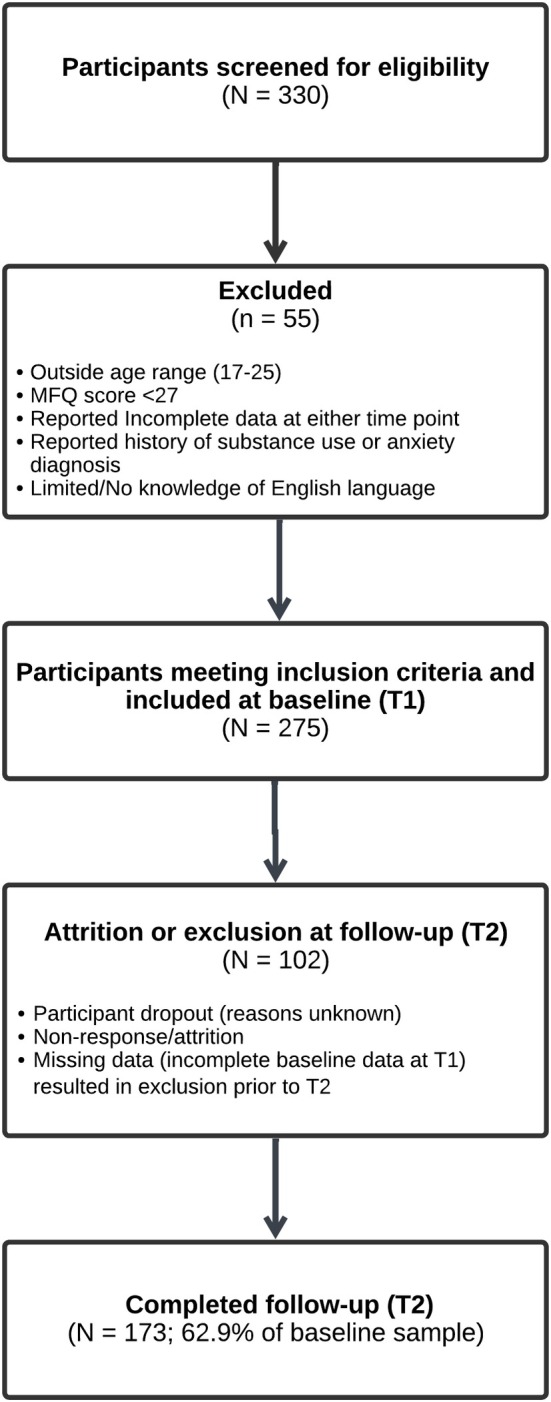
Participant flowchart outlining inclusion and exclusion criteria. **Note**. Participants were included based on the MFQ scores of ≥27 (Wood et al., 1995), a validated threshold for clinically significant depressive symptoms. A formal clinical diagnosis was not required for inclusion. This symptom‐based, dimensional approach allowed us to capture young people who may be undiagnosed or not currently engaged with mental health services. The sample reflects a population highly relevant to early intervention and aligns with transdiagnostic, inclusive research frameworks. A score of ≥27 indicates significant distress but does not equate to a confirmed diagnosis of MDD. A priori power analysis using G*Power (*F*‐test, linear multiple regression, medium effect size *f* ^2^ = .15, *α* = .05, power = .80, eight predictors) indicated a minimum sample size of 109 participants; the actual sample size exceeded this requirement.

Participants (N = 275) met the following inclusion criteria: (i) aged 17–25 years; (ii) met the clinical threshold for depressive symptoms (MFQ ≥ 27; Costello & Angold, 1988), indicating clinically significant symptoms of MDD; (iii) able to provide informed consent; and (iv) able to complete the research in English. It is important to note that, while these participants met the threshold for clinically significant symptoms based on self‐report, we do not know which participants had a formal clinical diagnosis of MDD. Exclusion criteria were: (i) not within the specified age range; (ii) MFQ score < 27; (iii) incomplete data at either time point; and (iv) reported a history of substance use or anxiety disorder diagnosis. These criteria ensured the sample reflected experiences primarily related to MDD, consistent with the study's aims. No additional exclusion criteria were applied (Figure 1).

### Participant recruitment procedure

Participants were recruited from local schools and the student population via online advertisements and posters. They were compensated with entry into a £50 Amazon voucher draw. Participants who consented to take part in the follow‐up phase were contacted via email approximately four months after the initial data collection. Of these participants, 173 provided follow‐up data. To reimburse their effort at follow‐up, they were entered into a further draw for one of five £50 Amazon vouchers.

### Participant attrition and follow‐up retention

A total of 102 participants could not be contacted for follow‐up assessments, had discontinued the study, or provided incomplete data. The decrease in sample size at follow‐up (N = 173) was mainly due to two factors: participant attrition and non‐response. Some individuals withdrew from the study, though the reasons for withdrawal were unknown. Additionally, several participants who completed the baseline assessment could not be reached for the follow‐up. Despite this reduction, demographic characteristics and the average scores for key variables were comparable between baseline and follow‐up. However, participants at follow‐up had, on average, lower levels of depressive symptoms (see Table 1 for further details).

**TABLE 1:** Participant Socio‐Demographic and Descriptive Characteristics at T1 and at T2.

Descriptive Characteristics	Mean (SD)		Range
	T1 (N = 275)	T2 (N = 173)	
Age	20.50 (2.19)	20.36 (20.03)	17–25
**Depression** **(MFQ)**	38.86 (9.26)	27.49 (14.50)	0–66
**Anhedonia Total Score** **(ASA)**	22.09 (7.97)	20.49 (8.65)	0–42
**Anhedonia** **Subscale** **1 (ASA‐S1)** *Enjoyment, Excitement, and Emotional Flattening*	9.45 (4.59)	8.50 (4.97)	0–21
**Anhedonia** **Subscale** **2 (ASA S2)** *Enthusiasm, Connection, and Purpose*	5.73 (1.86)	5.60 (1.89)	0–9
**Anhedonia** **Subscale** **3 (ASA‐S3)** *Effort, Motivation, and Drive*	6.92 (2.88)	6.39 (2.85)	0–12
**Loneliness (UCLA)**	51.35 (11.10)	49.80 (12.16)	0–80

Demographic Characteristics	N (%)	
	T1 (N = 275)	T2 (N = 173)
**Gender**		
Female	183 (66.50)	120 (43.60)
Male	82 (29.80)	48 (17.50)
Other	7 (2.50)	4 (1.50)
Prefer not to say	3 (1.10)	1 (.40)
**Ethnicity**		
Arabic	10 (3.60)	6 (2.20)
Asian	49 (17.80)	33 (12)
Black/African American	17 (6.20)	11 (4)
Hispanic/Latino/Spanish	5 (1.80)	3 (1.10)
Mixed/Multiple ethnic background	26 (9.50)	13 (4.70)
Other racial‐ethnic group	5 (1.8)	—
White	163 (59.30)	107 (38.90)
**Country of** **Residence**		
India	4 (1.50)	4 (1.50)
Malaysia	8 (2.90)	5 (1.80)
South Africa	18 (6.50)	10 (3.60)
United Kingdom	203 (73.80)	135 (49.10)
United States of America	18 (6.50)	5 (1.80)
Other (>1%)	24 (90)	14 (5.40)
**Highest** **Level of Education**		
High school	22 (8)	10 (3.60)
University	253 (92)	163 (59.30)

**Note**. *Abbreviations*: ASA, Anhedonia Scale for Adolescents; ASA‐S1, Anhedonia Subscale 1 (Enjoyment, Excitement, and Emotional Flattening); ASA‐S2, Anhedonia Subscale 2 (Enthusiasm, Connection, and Purpose); ASA‐S3, Anhedonia Subscale 3 (Effort, Motivation, and Drive); MFQ, Mood and Feelings Questionnaire; *N*, sample size; SD, standard deviation; UCLA, UCLA Loneliness Scale.

### Study procedure

Participants were provided with a link to the online participant information sheet and consent form. After reviewing the information sheet, they were given the opportunity to ask any questions about the study via email. Subsequently, they completed the measures outlined below using the Jisc online survey platform. All study information and consent procedures were completed at the outset of the study, and no additional consent was required for the follow‐up assessment.

### Sample size adequacy and G*Power

The sample size was determined based on statistical power calculations to ensure sufficient power for detecting associations between anhedonia, depression, and loneliness. A priori G*Power analysis was used to calculate the minimum sample size needed for the present study. We selected the *F*‐test, Linear Multiple Regression: Fixed model *R* ^2^ deviation from zero, with a medium effect size (*f* ^2^ = .15), 80% power, and an alpha value of (*α* err prob. = .05) with eight predictors to include covariates, which indicated we needed a total sample size of 109 participants.

### Data collection

#### Socio‐demographic characteristics

Participants provided socio‐demographic information at baseline (T1), including age, gender, ethnicity, country of residence, and highest level of education attained. Descriptive statistics for these variables are presented in Table 1.

#### Measures

Data were collected at both baseline (T1) and again at four month follow‐up (T2) for all measures included in the study.

##### Mood and Feelings Questionnaire (MFQ)

The MFQ is used to assess depressive symptoms (Costello & Angold, 1988). It consists of 33 items assessing symptoms experienced over the past two weeks, with participant responses reflecting thoughts, emotions, and behaviours rated on a three‐point scale (0 = *not true*, 1 = *sometimes true*, 2 = *true*). The scale is unidimensional, with total scores ranging from 0 to 66, determined by the sum of all items. Higher scores indicate greater levels of depressive symptoms. It has been validated in clinical trials and is suitable for adolescents (Daviss et al., 2006; Jarbin et al., 2020), and demonstrates excellent internal reliability (*α* = .91 to .93). A suggested cut‐off score of 27 has been shown to be optimal, offering the greatest diagnostic confidence when distinguishing clinical from non‐clinical populations, based on the intersection point of sensitivity [.78 (95% CI, .67 to .89)] and specificity [.78 (95% CI, .66 to .89)] (Wood et al., 1995). This score also indicates clinically serious depression (Wood et al., 1995). It has sufficient validity and reasonable diagnostic accuracy at this threshold (Thabrew et al., 2018). All participants completed the MFQ as a pre‐screening measure for depressive symptoms as part of the inclusion criteria, as well as for depression score analyses.

##### Anhedonia Scale for Adolescents (ASA)

The ASA was developed by our research group in collaboration with adolescents aged 12–18 experiencing symptoms of depression and anhedonia (Watson et al., 2021). The scale consists of 14 items rated on a four‐point scale (0 = *never*, 1 = *sometimes*, 2 = *often*, 3 = *always*), with higher scores indicating greater levels of anhedonia. Total scores range from 0 to 42. The ASA has three subscales: ASA‐S1 (Enjoyment, Excitement, and Emotional Flattening); ASA‐S2 (Enthusiasm, Connection, and Purpose); and ASA‐S3 (Effort, Motivation, and Drive). It has demonstrated high internal reliability, with a Cronbach's alpha of .94 for the total scale and subscale alphas ranging from .79 to .92. Test–retest reliability was also high (ICC = .73).

##### UCLA Loneliness Scale (UCLA)

The UCLA is a 20‐item general measure of subjective feelings of loneliness and social isolation, with responses rated on a four‐point Likert scale (1 = *never*, 2 = *rarely*, 3 = *sometimes*, 4 = *often*) (Russell, 1996). Total scores range from 0 to 80, with higher scores indicating greater loneliness. The scale has been found to have satisfactory psychometric properties, with good reliability and factorial validity (*α* = .89 to .94) (Link et al., 1991; Russell et al., 1978). It is widely used in both clinical and non‐clinical populations and has demonstrated sensitivity in detecting changes in loneliness over time.

### Data analysis

Data were analysed using the latest version of IBM SPSS Statistics (version 29). We examined the internal reliability of the ASA using Cronbach's alpha (*α*) and its test–retest reliability using intraclass correlation coefficients (ICC) from T1 to T2. Pearson correlations (*r*) were calculated to assess simple linear relationships between anhedonia and MDD and anhedonia and loneliness at T1.

We then conducted multiple linear regressions to test four models: (i) ASA total and subscales—ASA‐S1 (Enjoyment, Excitement, and Emotional Flattening); ASA‐S2 (Enthusiasm, Connection, and Purpose); and ASA‐S3 (Effort, Motivation, and Drive)—at T1 predicting MDD at T1, controlling for ethnicity and gender; (ii) ASA total and subscales at T1 predicting loneliness at T1, controlling for ethnicity, gender, and MDD at T1; (iii) ASA total and subscales at T1 predicting MDD at T2, controlling for ethnicity, gender, and MDD at T1; and (iv) ASA total and subscales at T1 predicting loneliness at T2, controlling for ethnicity, gender, MDD at T1, and loneliness at T1.

ASA total scores were analysed separately from the subscales—ASA‐S1 (Enjoyment, Excitement, and Emotional Flattening); ASA‐S2 (Enthusiasm, Connection, and Purpose); and ASA‐S3 (Effort, Motivation, and Drive)—which were examined together in the same model.

## RESULTS

### Participant characteristics and symptom assessment

Participants (N = 275) had a mean age of 20.50 years (SD = 2.19). Symptoms of MDD and anhedonia were assessed at two time points: baseline (T1) and four‐month follow‐up (T2) (see Table 1 for detailed information).

### ASA internal consistency reliability (Cronbach's alpha)

We found the ASA total score had internal reliability: *α* = .90 at T1 and *α* = .93 at T2. ASA‐S1 (Enjoyment, Excitement, and Emotional Flattening): *α* = .86 at T1 and *α* = .91 at T2; ASA‐S2 (Enthusiasm, Connection, and Purpose): *α* = .68 at T1 and *α* = .72 at T2; and ASA‐S3 (Effort, Motivation, and Drive): *α* = .84 at T1 and *α* = .85 at T2.

### ASA test–retest reliability (intraclass correlation coefficients, ICC)

The ASA demonstrated high temporal reliability (test–retest) for the total scale (ICC = .83 [.776, .877], *p* < .001), ASA‐S1 (Enjoyment, Excitement, and Emotional Flattening) (ICC = .80 [.733, .853], *p* < .001), ASA‐S2 (Enthusiasm, Connection, and Purpose) (ICC = .70 [.589, .774], *p* < .001), and ASA‐S3 (Effort, Motivation, and Drive) (ICC = .78 [.703, .837], *p* < .001).

### Pearson correlations (*r*)

Pearson correlations revealed that the ASA total score was correlated at T1 and T2 (*r* = .72). ASA‐S1 (Enjoyment, Excitement, and Emotional Flattening) was correlated at T1 and T2 (*r* = .67), ASA‐S2 (Enthusiasm, Connection, and Purpose) was correlated at T1 and T2 (*r* = .53), and ASA‐S3 (Effort, Motivation, and Drive) was correlated at T1 and T2 (*r* = .64). All correlations were significant at *p* < .01, two‐tailed (see Table 2).

**TABLE 2:** Bivariate Correlational Analysis Between Anhedonia, Depression, and Loneliness at T1 and T2.

Variables		T1						T2					
		1	2	3	4	5	6	7	8	9	10	11	12
**T1**	**1. Depression (MFQ)**	1											
	**2. Loneliness (UCLA)**	.45**	1										
	**3. Anhedonia Subscale 1 (ASA‐S1)** *Enjoyment, Excitement, and Emotional Flattening*	.67**	.63**	1									
	**4. Anhedonia Subscale 2 (ASA‐S2)** *Enthusiasm, Connection, and Purpose*	.24**	.41**	.38**	1								
	**5. Anhedonia Subscale 3** **(ASA‐S3)** *Effort, Motivation, and Drive*	.58**	.57**	.78**	.35**	1							
	**6. Anhedonia Total Score (ASA)**	.65**	.67**	.96**	.58**	.89**	1						
**T2**	**7. Depression (MFQ)**	.58**	.50**	.64**	.40**	.58**	.65**	1					
	**8. Loneliness (UCLA)**	.39**	.81**	.50**	.41**	.44**	.53**	.56**	1				
	**9. Anhedonia Subscale 1 (ASA‐S1)** *Enjoyment, Excitement, and Emotional Flattening*	.47**	.52**	.67**	.47**	.55**	.68**	.81**	.64**	1			
	**10. Anhedonia Subscale 2 (ASA‐S2)** *Enthusiasm, Connection, and Purpose*	.24**	.40**	.42**	.53**	.39**	.49**	.48**	.45**	.50**	1		
	**11. Anhedonia Subscale 3 (ASA‐S3)** *Effort, Motivation, and Drive*	.46**	.46**	.61**	.47**	.64**	.67**	.81**	.55**	.84**	.50**	1	
	**12. Anhedonia Total Score (ASA)**	.47**	.53**	.68**	.54**	.61**	.72**	.84**	.65**	.96**	.67**	.92**	1

** Correlation is significant at the .01 level (two‐tailed).

### Cross‐sectional data

#### Relationships between anhedonia, MDD, and loneliness

At baseline, Pearson correlations revealed that the ASA total score and its subscales—ASA‐S1 (Enjoyment, Excitement, and Emotional Flattening); ASA‐S2 (Enthusiasm, Connection, and Purpose); and ASA‐S3 (Effort, Motivation, and Drive)—all correlated with MDD and loneliness at T1 and T2 (see Table 2).

### Multiple linear regression analyses

Refer to Table 3 for the results of the multiple regression analysis, demonstrating that anhedonia (ASA) at T1 predicts MDD and loneliness at both T1 and T2, while *controlling for ethnicity, gender, and baseline levels of MDD and loneliness*.

**TABLE 3:** Anhedonia at T1 as a Predictor of Depression and Loneliness at T1 and T2, *Controlled for Ethnicity, Gender, and T1 Depression and Loneliness*.

Predictors T1	Outcomes T1										Outcomes T2									
	Depression					Loneliness					Depression					Loneliness				
	β	SE	*t*	*p*	*r*	β	SE	*t*	*p*	*r*	β	SE	*t*	*p*	*r*	β	SE	*t*	*p*	*r*
**Anhedonia Subscale 1 (ASA‐S1)** *Enjoyment, Excitement, and Emotional Flattening*	.586	.15	7.77	<.001	.46	.397	.20	4.84	<.001	.45	.298	.35	2.69	.008	.47	−.006	.24	−.07	.945	.68
**Anhedonia Subscale 2 (ASA‐S2)** *Enthusiasm, Connection, and Purpose*	−.032	.24	−.66	.510	.46	.196	.30	3.95	<.001	.45	.121	.50	1.86	.064	.47	.099	.34	1.91	.058	.68
**Anhedonia Subscale 3 (ASA‐S3)** *Effort, Motivation, and Drive*	.153	.23	2.14	.034	.46	.176	.28	2.38	.018	.45	.136	.48	1.38	.169	.47	−.113	.32	−1.45	.150	.68
**Anhedonia** **Total Score (ASA)**	.655	.05	14.24	<.001	.43	.651	.08	10.85	<.001	.45	.485	.13	6.49	<.001	.47	−.031	.10	−.44	.659	.67

**Note**. *Models*: Anhedonia total scores (ASA) were run in separate models to ASA subscales: ASA‐S1 (Enjoyment, Excitement, and Emotional Flattening), ASA‐S2 (Enthusiasm, Connection, and Purpose), and ASA‐S3 (Effort, Motivation, and Drive). T1 = Time 1 (baseline), T2 = Time 2 (four‐month follow‐up). Baseline sample size: N = 275; Follow‐up sample size: N = 173. *Control variables:* All models were controlled for ethnicity and gender, and depression at T1. Loneliness at T1 was controlled for when predicting loneliness at T2. *Outcome variables:* Depression at T1 and T2, and loneliness at T1 and T2 (listed in the top column). *Predictor variables:* Anhedonia total score (ASA) and its subscales ASA‐S1 (Enjoyment, Excitement, and Emotional Flattening), ASA‐S2 (Enthusiasm, Connection, and Purpose), and ASA‐S3 (Effort, Motivation, and Drive) (listed in the left‐hand column). *R* ^2^ values represent the proportion of variance in the dependent variable explained by the independent variables.

Refer to Table S1 for the results of the control variables used in the multiple regression analysis. Anhedonia (ASA) and its sub‐components at T1 predict depression and loneliness at both T1 and T2.

### Cross‐sectional data

#### The association between anhedonia and MDD at T1, *controlled for ethnicity and gender*

ASA total score predicted MDD at T1 [β = .655 (95% CI, .655 to .865), *p* < .001]. ASA‐S1 (Enjoyment, Excitement, and Emotional Flattening) [β = .586 (95% CI, .855 to 1.435), *p* < .001] and ASA‐S3 (Effort, Motivation, and Drive) [β = .153 (95% CI, .039 to .949), *p* = .034] predicted MDD at T1 (see Table 3 and Table S1).

#### The association between anhedonia and loneliness at T1, *controlled for ethnicity, gender, and MDD at T1*

ASA total score predicted loneliness at T1 [β = .651 (95% CI, .741 to 1.070), *p* < .001]. ASA‐S1 (Enjoyment, Excitement, and Emotional Flattening) [β = .397 (95% CI, .569 to 1.352), *p* < .001], ASA‐S2 (Enthusiasm, Connection, and Purpose) [β = .196 (95% CI, .585 to 1.751), *p* < .001], and ASA‐S3 (Effort, Motivation, and Drive) [β = .176 (95% CI, .118 to 1.238), *p* = .018] all predicted loneliness at T1 (see Table 3 and Table S1).

### Longitudinal data

#### The effects of anhedonia at T1 on MDD at T2, *controlled for ethnicity, gender, and MDD at T1*

ASA total score [β = .485 (95% CI, .590 to 1.107), *p* < .001], and ASA‐S1 (Enjoyment, Excitement, and Emotional Flattening) [β = .298 (95% CI, .249 to 1.631), *p* = .008] predicted MDD at T2. There was a trend for ASA‐S2 (Enthusiasm, Connection, and Purpose) [β = .121 (95% CI, −.056 to 1.930), *p* = .064] to predict MDD at T2 (see Table 3 and Table S1).

#### The effects of anhedonia at T1 on loneliness at T2, *controlled for ethnicity, gender, MDD at T1, and loneliness at T1*

ASA‐S2 (Enthusiasm, Connection, and Purpose) predicted loneliness scores at T2 at trend level [β = .099 (95% CI, −.021 to 1.314), *p* = .058] (see Table 3 and Table S1).

## DISCUSSION

This is the first study to examine the relationship between the sub‐components of anhedonia—ASA‐S1 (Enjoyment, Excitement, and Emotional Flattening); ASA‐S2 (Enthusiasm, Connection, and Purpose); and ASA‐S3 (Effort, Motivation, and Drive)—using the ASA scale, and clinically significant symptoms of MDD and loneliness in young people at both baseline (T1) and four‐month follow‐up (T2). We found that anhedonia and its sub‐components were associated with MDD and loneliness at baseline. Longitudinally, anhedonia total scores on the ASA and ASA‐S1 (Enjoyment, Excitement, and Emotional Flattening) predicted MDD, whereas ASA‐S2 (Enthusiasm, Connection, and Purpose) predicted loneliness at trend level.

### Anhedonia and MDD

Expanding the examination of anhedonia using the ASA questionnaire to a slightly older group of young people aged 17–25 years, we find that the ASA demonstrates strong internal reliability at both baseline and follow‐up. This was evidenced by high Cronbach's alpha scores and test–retest reliability, based on intraclass correlation coefficients, similar to findings from our previous study in a younger cohort with depressive symptoms (aged 12–18 years) (Watson et al., 2021).

Our findings are consistent with previous longitudinal research on anhedonia predicting depression (Barkus & Badcock, 2019; Gabbay et al., 2015; Khazanov et al., 2020; Mathew et al., 2011; Serretti, 2023; Spijker et al., 2001; Tan et al., 2020; Vinckier et al., 2017; Winer et al., 2017). However, we extend previous work by examining anhedonia sub‐components as predictors. Specifically, we show that anhedonia can predict clinically significant symptoms of MDD over time and that this may be driven by deficits in Enjoyment, Excitement, and Emotional Flattening. Our recent study has shown that activity enjoyment is driven by increased anticipation in real‐time using ecological momentary assessment methods (Sahni & McCabe, 2025). Therefore, we suggest novel interventions for anhedonia that increase anticipation for rewarding events could combat depression and loneliness in young people. One possibility is the use of interventions that can increase anticipation for activities through imagery (Hallford et al., 2022).

### Anhedonia and loneliness

Previous research has shown that anhedonia is linked to loneliness (Barkus & Badcock, 2019; Yang et al., 2023). Furthermore, our qualitative study found that social withdrawal was a prominent concern in young people with anhedonia (Watson et al., 2020), and loneliness has been identified as a mediator between anhedonia and social function (Tan et al., 2020). Based on this framework, we hypothesised that anhedonia and its sub‐components might not only be associated with loneliness but could also predict increased loneliness over time.

Our results support our hypotheses, as we found that the ASA total scores and ASA subscales were all associated with increased loneliness at baseline. This aligns with research demonstrating that anhedonia can contribute to social withdrawal and exacerbate feelings of isolation (Kuehner, 2017). We extend these previous findings by showing that the anhedonia sub‐component of Enthusiasm, Connection, and Purpose predicts loneliness over time. These results suggest that individuals with low enthusiasm and a diminished sense of purpose and connection may be particularly vulnerable to loneliness. This insight aligns with theories emphasising disconnection from others and a lack of purpose as key factors in the experience of loneliness (Hawkley & Cacioppo, 2010; Prizeman et al., 2023, 2024). Given that this finding was a trend approaching significance (*p* = .058), we consider it an important area for future research to explore.

### Theoretical implications

The results of this study lend support to various theoretical models, particularly regarding anhedonia's role in both the onset and persistence of MDD and loneliness. The Cognitive‐Behavioural Model of Depression suggests that a lack of positive reinforcement from pleasurable experiences can exacerbate depressive symptoms and social disengagement (Brown, 2001; Lewinsohn et al., 1998), and the Social Signal Processing Theory proposes that a diminished ability to experience pleasure may lead individuals to withdraw from social interactions, intensifying feelings of loneliness (Cacioppo et al., 2006; Hawkley & Cacioppo, 2010).

We argued that a more comprehensive examination of anhedonia and its sub‐components was needed to reveal which aspects of anhedonia impede an individual's capacity to connect with others and engage in fulfilling activities, thus contributing to both clinically significant symptoms of MDD and feelings of social isolation (Ma et al., 2025). Here, we have shown in more detail the nature of anhedonia using the ASA, whose components map to the neurobiological aspects of reward function. We demonstrate that different aspects of anhedonia uniquely influence MDD and loneliness, consistent with the view that anhedonia is a multidimensional construct rather than a singular phenomenon.

### Limitations and future directions

While this study provides valuable insights into the role of anhedonia in predicting clinically significant symptoms of MDD and loneliness among young people, several limitations should be considered. First, although we controlled for demographic variables such as ethnicity and gender, other potentially relevant factors—including family history of mental health conditions, socioeconomic status, formal depression diagnosis, and/or coping strategies—could have been incorporated as additional covariates. The reliance on self‐reported data may introduce bias; therefore, future studies would benefit from using multi‐informant methods or incorporating daily diary measures to better capture the ecological impact of anhedonia on mental health. Further research is needed to replicate these findings, particularly regarding the longitudinal effects of anhedonia on loneliness.

## CONCLUSIONS

This study highlights the important role of anhedonia and its sub‐component processes in predicting clinically significant symptoms of MDD and loneliness, thereby supporting theoretical models that emphasise its central role in these mental health outcomes. Using both cross‐sectional and longitudinal data, we show that the impact of anhedonia on depression and loneliness persists over time.

## AUTHOR CONTRIBUTIONS

**Katie Prizeman:** Conceptualization; methodology; software; data curation; investigation; validation; formal analysis; visualization; writing – original draft; writing – review and editing; project administration; resources; supervision. **Ciara McCabe:** Conceptualization; methodology; supervision; writing – review and editing; validation.

## FUNDING INFORMATION

None.

## CONFLICT OF INTEREST STATEMENT

The authors declare no conflicts of interest.

## CONSENT FOR PUBLICATION

Not applicable.

## Supporting information


Table S1:

## Data Availability

De‐identified data are publicly available and can be accessed via the University of Reading's Research Data Archive: Prizeman et al. (2025): Data supporting: ‘Anhedonia and its sub‐component processes predict clinically significant symptoms of Major Depressive Disorder (MDD) and loneliness in young people’. University of Reading. Data set. https://doi.org/10.17864/1947.001400.
