# Supplementary material for: Anhedonia and its sub‐component processes predict clinically significant symptoms of Major Depressive Disorder (MDD) and loneliness in young people
Source: Br J Clin Psychol. 2025 Aug 14;65(1):143–59. doi: 10.1111/bjc.70008 (PMC12889207; doi:10.1111/bjc.70008)
Supplement: Supplementary file 1 — Table S1: [file BJC-65-143-s001.pdf]

Table S1. Control Variables for Multiple Regression Analysis of Anhedonia and Its Subcomponent Processes at T1 as a Predictor of Depression and Loneliness at T1 and T2.

		**Outcomes T1**										**Outcomes T2**									
		**Depression**					**Loneliness**					**Depression**					**Loneliness**				
	**Controls T1**	β	*se*	*t*	*p*	*r*	β	*se*	*t*	*p*	*r*	β	*se*	*t*	*p*	*r*	β	*se*	*t*	*p*	*r*
**Anhedonia Subscales 1-3**	**Depression**	-	-	-	-	-	.032	.07	.52	.605	.45	.252	.13	3.21	.002	.47	.073	.08	1.18	.239	.68
	**Loneliness**	-	-	-	-	-	-	-	-	-	-	-	-	-	-	-	.804	.06	13.71	<.001	.68
	**Ethnicity**	-.002	.20	-.05	.964	.46	-.044	.23	-.97	.333	.45	.073	.37	1.29	.200	.47	-.024	.25	-.54	.593	.68
	**Gender**	-.027	.71	-.60	.549	.46	.037	.86	.81	.418	.45	.055	1.49	.97	.335	.47	.045	.99	1.00	.318	.68
**Anhedonia Total Score**	**Depression**	-	-	-	-	-	.023	.072	.39	.697	.45	.253	.12	3.39	<.001	.47	.048	.08	.81	.420	.67
	**Loneliness**	-	-	-	-	-	-	-	-	-	-	-	-	-	-	-	.809	.06	13.66	<.001	.67
	**Ethnicity**	-.019	.20	-.42	.675	.43	-.044	.23	-.96	.338	.45	.073	.37	1.30	.195	.47	-.017	.25	-.38	.702	.67
	**Gender**	-.001	.72	-.02	.982	.43	.037	.85	.80	.422	.45	.058	1.47	1.03	.303	.47	.048	.99	1.08	.283	.67

Models: Anhedonia total scores (ASA) were run in separate models to ASA subscales: ASA-S1 (Enjoyment, Excitement, and Emotional Flattening); ASA-S2 (Enthusiasm, Connection, and Purpose); and ASA-S3 (Effort, Motivation, and Drive).

Note. T1 = Time 1 (baseline), T2 = Time 2 (four-month follow-up).
Baseline sample size: N = 275; Follow-up sample size: N = 173.
Control variables: Ethnicity, gender, depression at T1, and loneliness at T1.
Outcome variables: Depression at T1 and T2, and loneliness at T1 and T2 (listed in the top column).
Predictor variables: Anhedonia total score (ASA) and its subscales ASA-S1 (Enjoyment, Excitement, and Emotional Flattening); ASA-S2 (Enthusiasm, Connection, and Purpose); and ASA-S3 (Effort, Motivation, and Drive) (listed in the left-hand column).
R^2 values represent the proportion of variance in the dependent variable explained by the independent variables.
